# Effect of Segmental Thoracic Epidural Block on Pancreatitisinduced Organ Dysfunction: A Preliminary Study

**DOI:** 10.5005/jp-journals-10071-23123

**Published:** 2019-02

**Authors:** Asha Tyagi, Yash Raj Gupta, Shukla Das, Gargi Rai, Arun Gupta

**Affiliations:** 1,2 Department of Anesthesiology and Critical Care; University College of Medical Sciences and GTB Hospital, NCT of Delhi, India; 3,4 Department of Microbiology; University College of Medical Sciences and GTB Hospital, NCT of Delhi, India; 5 Department of Surgery; University College of Medical Sciences and GTB Hospital, NCT of Delhi, India

**Keywords:** Pancreatitis, Procalcitonin, Sepsis, Thoracic epidural block

## Abstract

**Background:**

This preliminary randomized controlled study evaluated effect of thoracic epidural block (TEB) on progression of acute pancreatitis induced organ dysfunction/failure.

**Materials and Methods:**

Patients with predicted severe acute pancreatitis, without contraindication to TEB were randomized to receive (group TE) or not receive a TEB (group NTE) (n = 16 each). For group TE, TEB was performed at T8-9 or T9-10 vertebral level, with infusion of ropivacaine (0.2%) along with fentanyl 2 µg/mL; in group NTE, intravenous morphine was used instead, both interventions titrated to NRS of <4. SOFA score was assessed daily till discharge from ICU, and aggregate SOFA calculated by summing worst scores for each of organ system during ICU stay as primary outcome measure. Other surrogate measures of patient outcome were recorded as secondary objectives.

**Results:**

Aggregate SOFA score was statistically similar between both groups (group NTE: 3 [2 - 4]; group TE: 5 [2 - 6]) (*P* = 0.379); but there was trend of improvement in SOFA score in group TE versus a worsening in group NTE. Duration of hospital stay, and number of patients requiring mechanical ventilation were statistically similar; mortality was insignificantly lesser for group TE (12.5% versus 6.6%; *p* = 1.000). Fall in serum procalcitonin was significantly greater for group TE.

**Conclusion:**

Thoracic epidural was associated with insignificant clinical trend towards better organ functions and lesser mortality; along with significantly greater fall in serum procalcitonin. These are encouraging results that could guide future use of thoracic epidural in acute pancreatitis for its non-analgesic benefits.

**How to cite this article:**

Tyagi A, Gupta YR *et al.* Effect of Segmental Thoracic Epidural Block on Pancreatitis Induced Organ Dysfunction: A Preliminary Study. Indian J of Crit Care Med 2019;23(2):89-94.

## INTRODUCTION

**A**cute pancreatitis is characterized by inflammation of the pancreas that can progress to its necrosis, as well as systemic sepsis and multiple organ failure^1^. In severe form the mortality may approach up to 30% and organ dysfunction/failure remains a major component of the illness ^1, 2^.

Pathophysiology of pancreatitis involves an alteration in pancreatic microvascular perfusion and consequent derangements of its oxygenation ^3^. Role of thoracic epidural block in severe acute pancreatitis has been evaluated in experimental models, wherein it improved pancreatic microcirculation and oxygenation, decreased tissue damage, raised oxygenation and increased survival; probably as a consequence of increasing the splanchnic blood flow^4–7^. A recent clinical study also demonstrated thoracic epidural blockade to increase arterial perfusion of the pancreas and improve clinical outcome in patients with acute pancreatitis^8^.

Potential of thoracic epidural to retard progression and improve outcome in patients of pancreatitis presents an attractive area for further research ^9^. This preliminary randomized controlled trial was designed to evaluate role of thoracic epidural on progression of acute pancreatitis in patients with predicted severe form. The primary objective was to evaluate its effect on organ dysfunction/ failure, since it is a determinant of the severity of pancreatitis and clinical outcome.^10^

## MATERIALS AND METHODS

This trial was conducted in multidisciplinary ICU of a 1600-bedded hospital after approval by the Institutional Ethics Committee in meeting held on 24.10.14 and obtaining informed written consent from all participants. It is registered retrospectively with the Clinical Trial Registry of India (Number: CTRI/2015/08/006106; 18.08.2015). *Patient selection:* Patients diagnosed by the surgeon to have acute pancreatitis were enrolled and examined daily from day of admission onwards. Those with predicted severe pancreatitis were enrolled in the trial; evidenced by presence of any of the following: SIRS on admission or 48 hours later, obesity, clinical suspicion of severity, or Acute Physiology And Chronic Health Evaluation (APACHE) II score >8 within 48 hours after admission ^2,11^. The diagnosis of acute pancreatitis by the surgeon was based on the guidelines of the working group of International Association of Pancreatology (IAP)/American Pancreatic Association (APA)^2^.

Patients who refused consent, or had a contraindication to the thoracic epidural block, i.e., hemodynamic instability, skin infection at site of epidural catheter insertion, history of sensitivity to local anesthetics or spinal disease, coagulation abnormalities diagnosed by decreased platelet count (<100,000/mm^3^) and/ or increased International Normalized Ratio (INR >1.5); or had a clinically significant pleural effusion were excluded.

*Intervention:* Patients were randomized to one of two groups depending on institution or non-institution of thoracic epidural block (group TE or group NTE respectively). Block randomization was done through computer-generated table in groups of 4 patients each.

Those randomized to group TE were shifted to the operating room for performance of thoracic epidural block prior to ICU admission. In the operating room, non-invasive oscillometric blood pressure, lead II electrocardiography and pulse oximetry were instituted and intravenous access secured through which 5ml/kg of Ringer's lactate was infused as co-load. The epidural block was then performed under all aseptic precautions with patient in sitting position, using an 18-G Tuohy needle to locate the epidural space at T8-9 or T9-10 inter-vertebral level via midline approach with loss of resistance to air technique. The epidural catheter (Portex®; Smiths Medical; Czech Republic) was inserted 3-4 cm into epidural space and fixed in-situ. Ropivacaine (0.2%) was injected in aliquots of 2-3 mL till adequate pain relief i.e., numerical rating score (NRS <4). On shifting to the ICU, infusion of ropivacaine (0.2%) along with fentanyl 2 µg/mL was initiated using a syringe infusion pump (Medima®; Medima Ltd Al; Poland) at a rate titrated to maintain NRS <4. It was continued till required for pain relief or maximum duration of 96 hours following ICU admission. The epidural catheter was removed irrespective of duration in-situ, if any contraindication to its continued use developed at any time. Intravenous fluids and/ or ephedrine boluses were used to treat transient hypotension associated with the block.

Patients in group NTE did not receive the thoracic epidural and analgesia was provided by intravenous boluses of morphine (0.1-0.15 mg.kg ^-1^) titrated to the NRS of <4.

*Management in ICU:* The management was undertaken by a multidisciplinary approach involving the anesthesiologist, surgeon, and the radiologist as routinely done for patients of acute pancreatitis admitted to ICU.

Patients were discharged from ICU once no specific organ support was required and they could be cared for in ward, and the epidural infusion had been discontinued for group TE patients. *Outcome Measures:* The sequential organ failure assessment (SOFA) score is previously validated to quantify organ dysfunction/failure in critically ill patients including those with pancreatitis^12–14^. We chose aggregate SOFA score as the primary outcome measure that is calculated by summing the worst scores for each of the organ system during ICU stay^15^. For this, the SOFA score was assessed at time of admission to ICU, and then daily till discharge from ICU.

Secondary outcome measures included duration of hospitalization (from admission to discharge/death from hospital), need of mechanical ventilation and the in-hospital mortality.

Other characteristics noted for comparison of the two groups included age of the patient, cause of pancreatitis, duration of illness prior to hospital admission, route of feeding, surgical intervention, and radiologic findings when available, specifically including evidence for development of pancreatic necrosis.

In a small subset of patients, change in certain inflammatory mediators after 2 days of the intervention was noted. Blood sample was collected at time of inclusion in the study and two days later for assessment of procalcitonin, C-reactive protein (CRP), Tumor necrosis factor-α (TNF-α) and Interleukin (IL)-6 (group TE: 7 patients; and group NTE: 6 patients). The sample of blood was collected aseptically at both of the predefined time points and allowed to stand at room temperature for 1 hour to clot. The supernatant was removed and placed in new tube. Serum was stored at −80°C till further use. For the assay, serum was seeded on a 96-welled plate, and procalcitonin (Biovendor,® Czech Republic), CRP (DRG,® Germany), TNF-α and IL-6 (Diaclone,® France) measured by commercially available ELISA according to manufacturers instruction with minimum detection limit of 10 ng/mL, 15 pg/mL, 8 pg/mM and 2 pg/mL respectively.

*Blinding:* Presence of epidural catheter in patients of group TE precluded blinding to group allocation. The parameters required for calculation of primary outcome measure (SOFA score) were however recorded by an anesthesiologist who was uninvolved in the study. *Sample Size:* There is no published trial regarding effect of segmental thoracic epidural block on pancreatitis induced organ dysfunction in terms of SOFA score. This was a preliminary study that included 16 patients in each group. In one patient randomized to group TE, there was a failure to institute the block and thus the statistical analysis was done for 16 patients of group NTE and 15 of group TE. *Statistical Analysis:* Since this is a pilot study we have reported results as descriptive statistics. As recommended for pilot studies, *p* value <0.2 was considered as statistically significant^16^. For the primary outcome measure, besides hypothesis testing we also calculated the standard error (CI) of the difference in means at various CIs. We assumed the minimum clinically important difference in the aggregate SOFA score to be −2 for interpretation of the descriptive CIs.

## RESULTS

Total of 88 patients with acute pancreatitis were evaluated for inclusion (Flowchart 1).

### Organ Functions

Despite randomization, the SOFA score was worse for group TE as compared to group NTE at time of admission (Table 1).

Aggregate SOFA score was clinically higher but statistically similar between group TE and group NTE (5 [2 - 6] vs. 3 [2 - 4]; *p* = 0.379).

The difference in means of aggregate SOFA score between the two groups was 0.4, and the standard error (95% CI) was −1.7 (-3 to 3.9). Since this CI crosses zero as well as the assumed MCID of −2, the difference in means of aggregate SOFA is not a result of the intervention being evaluated. We also calculated the various CIs ranging between 95% to 60%: 90% CI: −2.4 to 3.3; 85% CI: −2 to 2.9; 75% CI: −1.5 to 2.4; and 60% CI: −1 to 1.8. Since all the CIs crossed zero, with or without crossing the MCID, there appears to be no clinically important difference in the aggregate SOFA scores between group NTE and group TE even at lower CIs.

**Graph 1:**
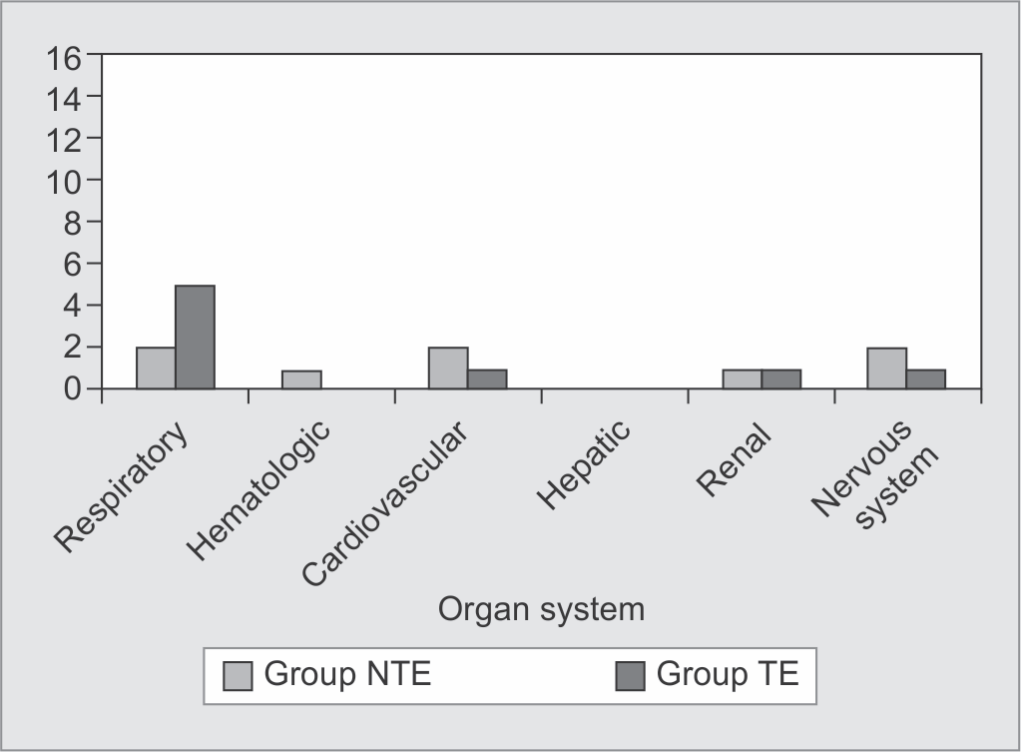
Intergroup comparison of individual organ system failure

There was a clinical trend towards improvement of SOFA score for group TE as compared to group NTE. The daily SOFA score showed improvement for group TE and a worsening for group NTE over time (Table 1). Also, failure of cardiovascular, hematologic, central nervous and renal systems (defined as SOFA subscore of ≥3) was lesser for group TE (Graph 1). Failure of respiratory system on the other hand was greater for group TE as compared to group NTE. Amongst patients with respiratory failure however, the mortality as well as need for mechanical ventilation was lesser for group TE as compared to group NTE (20% vs. 100%).

**Flowchart 1:**
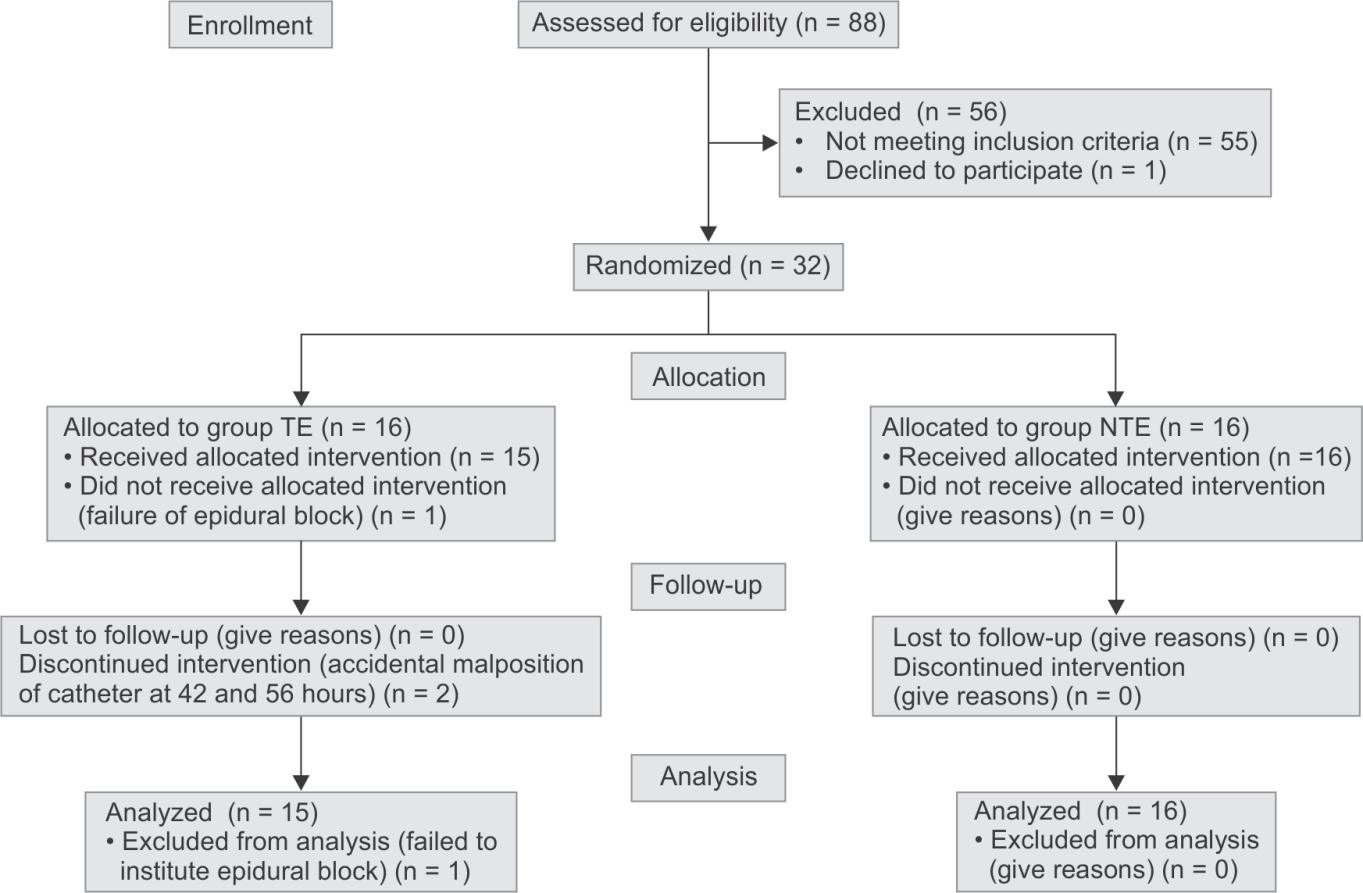
CONSORT flow chart

**Table 1:** Sepsis related organ failure assessment (SOFA) score and other markers of morbidity

Marker of morbidity	Group NTE	Group TE	P value
Duration of hospital stay	7 [5 - 8.7]	8 [7 - 13]	0.052
Mechanical ventilation	2 (12.5%)	2 (13.3%)	1.000
SOFA0	2 [2 - 4]	4 [2 - 5]	-
SOFA1	2 [1 - 4]	4 [1 - 5]	-
SOFA2	2 [1 - 14]	2 [0 - 4]	-
SOFA3	10 [3 - 16]	2 [1 - 6]	-
In-hospital mortality	2 (12.5%)	1 (6.6%)	1.000

Values are median [IQR] or number of patients (%). SOFA_Day_ of ICU stay.

### Secondary Outcome Measures

The in-hospital mortality was clinically lesser for group TE as compared to group NTE (6.6% vs. 12.5%) (*p* = 1.000).

The number of patients requiring mechanical ventilation was similar between both groups (Table 1) (*p* = 1.000). The duration of hospital stay was slightly longer with group TE as compared to group NTE (*p* = 0.052).

### Inflammatory Markers

Serum procalcitonin, TNF-*a*, IL-6 and CRP showed a decrease for group TE but not group NTE (Table 2). The decrease in serum procalcitonin was significantly greater for group TE (*p* = 0.010); for TNF- *a* and IL-6 also there was a clinically greater decrease for group TE as compared to group NTE, although it failed to achieve statistical significance (Table 2).

**Table 2:** Change in various inflammatory mediators^a^

*Parameters*	*Group NTE (n = 7)*	*Group TE (n = 6)*	*P-value*
Procalcitonin (ng/ml)	0 [-0.01-0.03]	0.6 [0.1 - 4.3]	0.010
C-reactive protein (mg/l)	4.6 [0.3 - 7.9]	3.5 [3.9 - 10.1]	1.000
Tumor necrosis factor-α (pg/ml)	-11 [-36 - 7]	14 [-75 - 114]	0.253
Interleukin-6 (pg/ml)	-4 [-10 - 23]	69 [-44 - 137]	0.391

^a^Change calculated after 2 days of intervention; positive values denote a decrease and negative values an increase. Values are median [IQR].

**Table 3:** Patient characteristics at time of ICU admission

*Characteristic*	*Group NTE (n = 16)*	*Group TE (n = 15)*	*p value*
Age (years)	32 [23-59]	40 [34-45]	0.513
Heart rate (bpm)	110 [104-116]	110 [110-116]	0.435
Temperature (°C)	37 [36.6-37]	37 [36.4-37]	0.299
Respiratory rate (/min)	24 [23-26]	26 [24-26]	0.046
Total leukocytic count (/mm^3^)	13,050 [10,400-15,325]	15,000 [13,500-18,200]	0.048
Mean arterial pressure (mm Hg)	100 [94-106]	98 [96-106]	0.968
Arterial pH	7.44 [7.37-7.47]	7.40 [7.40-7.50]	0.721
Serum creatinine (mg/dL)	0.9 [0.8-1.1]	0.9 [0.8-1.3]	0.326
Hematocrit (%)	36 [29.9-39.6]	37 [33.4-38.4]	0.373
Glasgow coma score	15 [15-15]	15 [15-15]	1.000
Serum potassium (mEq/L)	4 [3.7-4.2]	3.8 [3.5-4.4]	0.322
Serum sodium (mEql/L)	139 [136-142]	132 [131-138]	0.007
PaO_2_ (mm Hg)	73 [54-90]	64 [56-84]	0.429
Stay hospitalization prior to ICU admission (days)	1 [1-1.7]	2 [0-2]	0.337

Values are number of patients or median [IQR]. APACHE, Acute physiology and chronic health evaluation score.

### Other Patient Characteristics

The median age of patients was similar between group NTE and group TE (Table 3).

At the time of ICU admission, all the included patients had presence of SIRS and the APACHE II score was statistically similar between group TE and group NTE, although it was clinically greater in the former (7 [5-10 vs. 6 [3-10] respectively; *p* = 0.706).

Amongst parameters required for diagnosing presence of SIRS and calculating APACHE II at this time, the respiratory rate, total leucocytic count, and serum sodium were significantly worse (*p* < 0.2) and PaO_2_ clinically lower (*p* > 0.2) for group TE than group NTE (Table 3).

Duration of hospitalization prior to ICU admission was statistically similar between both groups (*p* > 0.2; Table 3).

### Ancillary Observations Related to Pancreatitis

The most common cause of acute pancreatitis was cholelithiasis for group NTE as well as group TE (13/16 vs. 8/15).

The serum amylase value at time of inclusion was 1200 [715-1200] and 929 [210 - 1200] IU/L, for group NTE and group TE respectively.

The serum lipase was 123 [96 - 194] and 360 [222 - 456] IU/L, for group NTE and group TE respectively.

Number of patients requiring total parenteral nutrition was 4/16 (25%) for group NTE and 2/15 (13%) for group TE.

None of the patients in either group underwent surgery for pancreatitis.

### Related to Thoracic Epidural

Median volume of ropivacaine (0.2%) infused through epidural catheter was 93 [54-110] mL per day for group TE. The median duration of epidural infusion was 72 [62-90] hours, ranging from 42 hours to 96 hours. Ephedrine was required for managing post-epidural hypotension in only 1/15 (6.6%) patient. There was no infective complication related to the thoracic epidural block. Morphine requirement for group NTE was 15 [10.3-15] mg per day.

Pain relief was present and adequate in all patients of group TE following the block.

## DISCUSSION

This was a pilot study that evaluated the effect of segmental thoracic epidural blockade on organ dysfunction/failure in predicted severe pancreatitis.

Using an *a* error of 0.2, thoracic epidural was not associated with an improvement in the aggregate SOFA score. The lack of effect on aggregate SOFA score should, however, be viewed keeping in mind allocation of sicker patients despite randomization to the group using thoracic epidural. The SOFA and APACHE II scores were worse and duration of hospitalization prior to ICU admission longer for the thoracic epidural intervention group, at time of inclusion into the study.

Despite the randomization bias, the use of thoracic epidural appeared to be associated with a clinical trend, although insignificant, of better recovery. There was an improvement in organ functions as assessed by the daily SOFA scores while absence of the thoracic epidural was accompanied not only by a lack of improvement of organ functions over time, but rather a worsening was evident. Also, almost all of the organ systems *viz* ., cardiovascular, hematologic, central nervous and renal showed decreased incidence of failure following use of thoracic epidural. Recovery from respiratory system failure was also better with thoracic epidural usage, there was decreased in-hospital mortality (6.6% vs 12%) and lesser requirement of parenteral nutrition (13% vs 26%). All of these findings suggest a possible potential for benefit of thoracic epidural in acute pancreatitis in adequately sized further trials. The change in systemic mediators also shows that thoracic epidural could indeed have a role in decreasing the inflammatory response of pancreatitis. It resulted in significantly greater fall in serum procalcitonin at 48 hours and an insignificant decrease in TNF-α and CRP as well. Serum procalcitonin is a well-known marker to grade severity and outcome of pancreatitis ^2,17^.

Benefits of thoracic epidural in pancreatitis are hypothesized to be due to sympathetic blockade induced increase in splanchnic circulation^7^. These are distinct from the analgesia or enhanced gastrointestinal motility that are previously well established with use of the block ^18^.

Pancreatitis has remained a disease with high mortality, and there is no specific intervention that could alter the prognosis. In the face of all such data, our findings suggest that it is warranted to conduct further randomized prospective trials evaluating effect of thoracic epidural on progression of pancreatitis. In a very recent propensity analysis also a mortality reduction with thoracic epidural block was shown for patients of acute pancreatitis, although no effect on organ functions was included ^19^.

For future trials, the outcome measures to evaluate benefits in patients with pancreatitis could be multiple. Besides organ functions, surrogate markers of outcome in patients of pancreatitis could include mortality, need for surgery, ICU admission, mechanical ventilation, duration of hospital or ICU stay, and need of parenteral nutrition. While we chose to analyze the organ functions, effect of thoracic epidural on arterial perfusion of the pancreatic gland evidenced by CECT imaging has also been studied^8^. We could not subject all patients to CECT scans for evaluation of pancreatic circulation due to logistic constraints, and imaging was done only when clinically indicated for either a diagnostic dilemma or worsening of the condition^20, 21^.

Another consideration for future trials would be to decide the patients of acute pancreatitis in whom to explore the potential for benefits. We included patients of acute pancreatitis with a predicted severe attack, representing a small and very specific subgroup of the disease presentation. Thoracic epidural block is a commonly used anesthetic intervention, but it is associated with its own set of adverse effects ^9^. To balance the risk-benefit profile of epidural block, we narrowed the inclusion criteria to only “predicted severe” cases. This excluded patients with mild form of acute pancreatitis that is known to be usually a self-limiting affliction, with organ dysfunction/failure being very rare. The highly selective subgroup in which results can be applied is evident from the fact that we obtained our sample size of 32 patients after evaluating 88 patients of acute pancreatitis. Future trials could be limited to “predicted severe” cases or include all severe acute pancreatitis patients instead.

At the time of initiation of this study evidence for successful and safe analgesic use of epidural in these patients existed^22–24^. However, possible risks associated with thoracic epidural in patients of pancreatitis were also commented upon^9^. The two major concerns could be the associated hypotension and infective neuraxial complications. An earlier reported incidence of hypotension following use of epidural in patients with acute pancreatitis was 37.5%^24^. However, we noted a much lower incidence of post-epidural hypotension (6.6%). The earlier high incidence of hypotension may be a result of the empiric large volumes of local anesthetic that were used in contrast to titrated and diluted local anesthetic in our study^24^. Use of titrated dilute concentration of local anesthetic along with opioid to decrease the requirement and judicious fluid and vasopressor administration could help to avoid hypotension.

We did not encounter any epidural related neuraxial infective complication. This can be due to the short duration of epidural catheterization (up to 96 hours), use of prophylactic antibiotic, or the absence of bacteremia itself. In a recent audit of use of epidural block in critically ill patients there was a very low incidence of infective neuraxial complications (0.8%) and that too in the presence of multiple epidural catheter placements and laboratory proven methicillin resistant *Staphylococcus aureus* bacteremia^22^. The duration of epidural anesthesia may be an important determinant of infective complications. It was noted to be 11 days (3 −38 days) in the audit that recorded occurrence of infective neuraxial complications with epidural catheterization in ICU, while in our study the median duration was much shorter viz., 72 (62-90) hours. The duration of 5.7 days was also noted to be safe in another report of use of thoracic epidural in patients with acute pancreatitis^8^.

Thus, thoracic epidural can be considered safe in patients of acute pancreatitis, when performed carefully and while adhering to its routine contraindications such as hemodynamic instability and coagulopathy.

To conclude, the present results are encouraging and show that there may be a beneficial role of thoracic epidural for selected patients with acute pancreatitis. The use of this routine anesthetic technique is safe in these patients, but demands careful implementation. Further research to evaluate and validate the beneficial role of thoracic epidural block in preventing the progression of the disease in patients of acute pancreatitis should be undertaken.
